# Substance P Serves as a Balanced Agonist for MRGPRX2 and a Single Tyrosine Residue Is Required for β-Arrestin Recruitment and Receptor Internalization

**DOI:** 10.3390/ijms22105318

**Published:** 2021-05-18

**Authors:** Chalatip Chompunud Na Ayudhya, Aetas Amponnawarat, Hydar Ali

**Affiliations:** 1 Department of Basic and Translational Sciences, School of Dental Medicine, University of Pennsylvania, Philadelphia, PA 19104, USA; chalatip@upenn.edu (C.C.N.A.); aetasa@upenn.edu (A.A.); 2 Department of Oral Diagnosis, Faculty of Dentistry, Naresuan University, Phitsanulok 65000, Thailand; 3 Department of Family and Community Dentistry, Faculty of Dentistry, Chiang Mai University, Chiang Mai 50200, Thailand

**Keywords:** mast cells, MRGPRX2, MrgprB2, tyrosine, substance P, signaling, internalization

## Abstract

The neuropeptide substance P (SP) mediates neurogenic inflammation and pain and contributes to atopic dermatitis in mice through the activation of mast cells (MCs) via Mas-related G protein-coupled receptor (GPCR)-B2 (MrgprB2, human ortholog MRGPRX2). In addition to G proteins, certain MRGPRX2 agonists activate an additional signaling pathway that involves the recruitment of β-arrestins, which contributes to receptor internalization and desensitization (balanced agonists). We found that SP caused β-arrestin recruitment, MRGPRX2 internalization, and desensitization. These responses were independent of G proteins, indicating that SP serves as a balanced agonist for MRGPRX2. A tyrosine residue in the highly conserved NPxxY motif contributes to the activation and internalization of many GPCRs. We have previously shown that Tyr^279^ of MRGPRX2 is essential for G protein-mediated signaling and degranulation. To assess its role in β-arrestin-mediated MRGPRX2 regulation, we replaced Tyr^279^ in the NPxxY motif of MRGPRX2 with Ala (Y279A). Surprisingly, we found that, unlike the wild-type receptor, Y279A mutant of MRGPRX2 was resistant to SP-induced β-arrestin recruitment and internalization. This study reveals the novel findings that activation of MRGPRX2 by SP is regulated by β-arrestins and that a highly conserved tyrosine residue within MRGPRX2’s NPxxY motif contributes to both G protein- and β-arrestin-mediated responses.

## 1. Introduction

Mast cells (MCs) are key sentinel immune cells that are often found in close proximity to sensory nerve endings in various tissues, including the skin, gastrointestinal mucosa, and respiratory tract [1]. The role of the MC–neuron interaction as a regulatory unit in both physiology and disease has recently gained prominence [2,3,4,5,6,7]. Substance P (SP) has long been established as an inflammatory neuropeptide and is known to activate a variety of cell types via the neurokinin-1 receptor (NK-1R). Mouse bone marrow-derived MCs (BMMCs) express NK-1R and its expression level is upregulated upon FcεRI stimulation [8]. However, emerging evidence suggests that SP contributes to neurogenic inflammation and pain in mice through MC activation via a novel G protein-coupled receptor (GPCR) known as Mas-related GPCR-B2 (MrgprB2; human ortholog MRGPRX2) [3,4]. Activation of MrgprB2 by SP is required for regulating inflammatory hyperalgesia via the release of pro-inflammatory cytokines and chemokines, as well as the recruitment of immune cells at the injury site, which facilitates inflammatory responses and peripheral sensitization [3]. Furthermore, Serhan et al. recently showed that SP released from nociceptors activates murine skin MCs and contributes to the development of atopic dermatitis, a type 2 allergic skin disease, through the activation of MrgprB2 [4]. These findings challenge our current understanding of SP-mediated neuroinflammatory diseases and pain, and raise an interesting possibility that targeting MRGPRX2 might represent a promising therapeutic approach for the management of these neuroinflammatory associated conditions in humans. However, the mechanisms involved in the activation and regulation of MRGPRX2 by SP have yet to be fully elucidated.

As a member of class A GPCR family, MRGPRX2 shares a common structure of seven transmembrane (TM) α-helices. The extracellular part is responsible for ligand binding, whereas the intracellular part is involved in binding downstream effectors such as heterotrimeric G proteins [9]. Besides G protein-mediated signaling, most GPCRs signal via an additional pathway that involves the recruitment of adapter proteins known as β-arrestins [10,11]. The recruitment of β-arrestins results in uncoupling of the receptor from G proteins and termination of receptor activation (desensitization) [11,12]. They further target the receptor internalization/endocytosis, which downregulates signaling as the receptor is physically removed from the cell surface [13,14,15]. In addition to receptor desensitization and internalization, it is now established that β-arrestins also play important roles in various G protein-independent downstream signaling to promote chemotaxis and to modulate inflammation [15,16,17]. Thus, β-arrestins regulate nearly all aspects of receptor activity, including desensitization, downregulation, trafficking, and signaling.

While most GPCR agonists target signaling pathways mediated by both G proteins and β-arrestins (“balanced agonists”), some agonists preferentially activate only particular pathway (“biased agonists”) [18,19]. GPCR agonists that preferentially activate G proteins are known as G-protein-biased and those that activate β-arrestins are known as β-arrestin-biased agonists. For MRGPRX2, compound 48/80 and codeine activate both G proteins and β-arrestins (balanced agonists) and can cause receptor internalization, which is associated with functional desensitization [20,21]. On the contrary, host defense peptides, such as cathelicidin LL-37 and angiogenic peptide AG-30/5C, activate only G proteins, but not β-arrestins (G protein-biased), and do not induce receptor desensitization and internalization [20,22,23]. Biased signaling has gained important therapeutic implications in several GPCRs [19]. However, whether SP acts as a balanced or biased agonist for MRGPRX2 remains unknown.

Closely related GPCRs exhibit a high degree of conserved sequence motifs and a common activation pathway, especially in the regions implicated in ligand binding and G protein coupling [24]. Recent computer-based structural modeling, sequence analysis, and mutagenesis studies have led to the identification of residues in MRGPRX2 that are responsible for ligand binding and G protein coupling [25,26,27,28]. One of the most conserved GPCR sequences is the NPxxY motif located in TM7. A tyrosine residue Tyr^7x53^ in this motif is pivotal for receptor activation for all class A GPCRs [29]. Structural modeling studies in β_2_ adrenergic receptor, rhodopsin, and M2 muscarinic acetylcholine receptors revealed that, upon ligand binding, Tyr^7x53^ undergoes substantial rotamer conformations and provides activation switch through the formation of a water-mediated hydrogen bond [30,31]. This favors an outward movement in the cytoplasmic end of TM6 and allows the receptor coupling to G proteins and other signal transducers, which represents a hallmark of GPCR activation. Besides its importance in receptor activation, Tyr^7x53^ is suggested to regulate agonist-induced receptor internalization in many GPCRs [32,33,34,35]. In MRGPRX2, the corresponding tyrosine residue is Tyr^279^. Our previous study has shown that this tyrosine residue is required for G protein-mediated MRGPRX2 activation in response to SP [28]. However, the role of this tyrosine residue on β-arrestin-mediated MRGPRX2 signaling has not been determined.

The goals of the current study were to determine whether SP serves as a balanced or biased agonist for MRGPRX2 and to investigate the potential effects of MRGPRX2’s Tyr^7x53^ (Tyr^279^) residue on β-arrestin recruitment and receptor internalization. The data presented herein suggest that SP serves as a balanced agonist for MRGPRX2 and that Tyr^7x53^ contributes to both G protein-dependent signaling for degranulation and G-protein independent signaling for β-arrestin recruitment and receptor internalization.

## 2. Results

### 2.1. SP Is a Balanced Agonist for MRGPRX2

**Figure 1:**
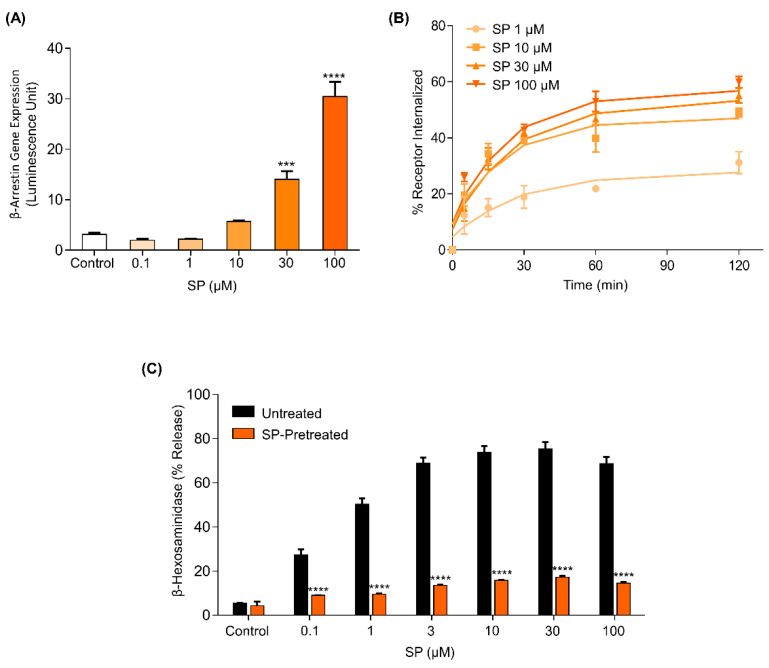
Substance P (SP) is a balanced agonist for MRGPRX2. (**A**) HTLA cells stably expressing MRGPRX2 (HTLA-MRGPRX2) were exposed to different concentrations of SP for 16 h, and β-arrestin–mediated gene expression was determined. (**B**) Rat basophilic leukemia (RBL) cells stably expressing MRGPRX2 (RBL-MRGPRX2) were stimulated with different concentrations of SP for the times indicated, and level of receptor internalization was determined by flow cytometry. (**C**) RBL-MRGPRX2 cells were cultured in the absence (untreated) or presence of SP (30 μM) for 16 h. Cells were subsequently stimulated with different concentrations of SP for 30 min, and percent degranulation was determined by β-hexosaminidase release assay. All data points are the mean ± SEM of at least three experiments. For comparisons of two samples, two-tailed unpaired *t*-test was used. For comparisons of multiple samples to a control group, one–way analysis of variance (ANOVA) with Dunnett’s post-hoc test was used. *** *p* < 0.001 and **** *p* < 0.0001.

For many GPCRs, biased signaling has important therapeutic implications as it can elicit distinct physiological responses [18,19,36]. Certain MRGPRX2 ligands, including compound 48/80, codeine, and HDP AG-30/5C, have recently been identified as either balanced or G-protein-biased agonists [20,21]. SP is a well-known MRGPRX2 agonist; however, whether SP is a balanced or biased agonist for MRGPRX2 has yet to be investigated. We have previously shown that SP induces Ca^2+^ mobilization and degranulation in a G protein-dependent manner [28]. Thus, we first sought to determine if SP can also trigger β-arrestin recruitment. For this, we utilized an assay known as transcriptional activation following arrestin translocation (TANGO) using HTLA cells (engineered HEK-293T cells stably expressing a β-arrestin2–tobacco etch virus fusion gene) stably expressing human MRGPRX2 (HTLA-MRGPRX2) [20,25]. Cells were exposed to either buffer (control) or SP at different concentrations for 16 h and β-arrestin-mediated gene expression (indicative of β-arrestin recruitment) was measured. We found that SP (30 μM and 100 μM) significantly induced β-arrestin-mediated gene expression (Figure 1A).

For most GPCRs, the recruitment of β-arrestins is involved in receptor internalization and desensitization. Compound 48/80 and codeine trigger robust β-arrestin recruitment, MRGPRX2 internalization, and inhibition of degranulation in response to subsequent stimulation by the same ligand (desensitization) [20,21]. Given that SP induced β-arrestin recruitment, we hypothesized that it could also cause receptor internalization and desensitization. Rat basophilic leukemia (RBL-2H3) cell line, a commonly used model for MC activation, does not endogenously express Mrgpr receptors [22,37]. We thus utilized RBL-2H3 cells stably expressing human MRGPRX2 (RBL-MRGPRX2) to determine the effects of SP on MRGPRX2 internalization and desensitization. For receptor internalization, RBL-MRGPRX2 cells were stimulated with different concentrations of SP for different time-points and incubated with PE-conjugated anti-MRGPRX2 antibody to determine cell surface receptor expression by flow cytometry. Consistent with β-arrestin recruitment, we found that SP triggered MRGPRX2 internalization and that this response was dose-dependent (Figure 1B). Furthermore, preincubation of RBL-MRGPRX2 with 30 μM of SP overnight resulted in nearly complete inhibition of degranulation on second stimulation by the same ligand (Figure 1C). Taken together, these studies suggest that SP serves as a balanced agonist for MRGPRX2.

### 2.2. SP-Induced β-arrestin Recruitment and MRGPRX2 Internalization Are G Protein-Independent

**Figure 2:**
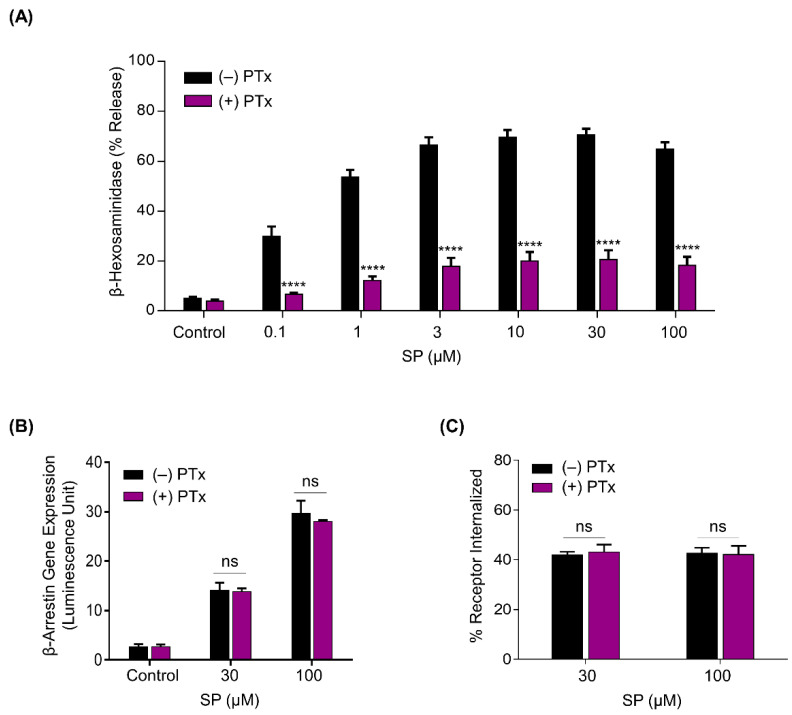
Pertussis toxin (PTx) inhibits SP-induced mast cell (MC) degranulation, but has no effect on β-arrestin recruitment and MRGPRX2 internalization. RBL-MRGPRX2 cells were cultured in the absence or presence of PTx (100 ng/mL, 16 h), and the effects of SP on (**A**) degranulation, (**B**) β-arrestin-mediated gene expression, and (**C**) MRGPRX2 internalization were determined. All data points are the mean ± SEM of at least three experiments. Statistical significance was determined by two-tailed unpaired *t*-test. **** *p* < 0.0001.

SP has been shown to induce MRGPRX2-mediated Ca^2+^ mobilization and MC degranulation in a G protein-dependent manner [28]. We thus asked if β-arrestin recruitment and MRGPRX2 internalization in response to SP are also mediated in a G protein-dependent manner. We utilized a G protein inhibitor, pertussis toxin (PTx), and confirmed that pretreatment of RBL-MRGPRX2 cells with PTx (100 ng/mL, 16 h) attenuated degranulation in response to SP (Figure 2A). By contrast, PTx had no effect on SP-induced β-arrestin-mediated gene expression and MRGPRX2 internalization (Figure 2B,C). These findings indicate that β-arrestin recruitment and MRGPRX2 internalization in response to SP are mediated independently of G proteins.

### 2.3. β-Arrestin2 Regulates SP/MrgprB2-Mediated MC Degranulation

**Figure 3:**
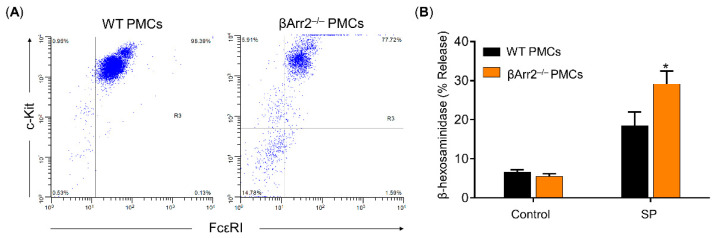
β-arrestin2 regulates MrgprB2-mediated MC degranulation in response to SP. (**A**) Peritoneal MCs (PMCs) obtained from wild type (WT) and βArr2^−/−^ mice displayed similar levels of surface c-Kit and FcεRI expression as determined by flow cytometry. (**B**) Cells were exposed to either buffer (control) or SP (50 μM) for 30 min, and β-hexosaminidase release was determined. All data points are the mean ± SEM of at least three experiments performed in triplicate. Statistical significance was determined by two-tailed unpaired *t*-test. * *p* < 0.05.

MrgprB2 has been identified as the mouse ortholog of human MRGPRX2 [38]. Previous studies showed that SP activates murine MCs to cause degranulation and inflammatory responses via MrgprB2 [3,38]. Of note, there are significant differences in agonist affinities between mouse MrgprB2 and human MRGPRX2 receptors. While SP activates MRGPRX2 with an EC_50_ of 152 nM, it activates MrgprB2 with a higher EC_50_ value of 54 µM [38]. To investigate the biological role of β-arrestin2 on SP/MrgprB2-mediated MC responses, we utilized peritoneal MCs (PMCs) obtained from wild-type (WT) and β-arrestin2 knockout (βArr2^−/−^) mice [39]. Absence of β-arrestin2 had no effect on the development and maturation of MCs, as shown by similar levels of cell surface FcεRI and c-Kit expression (Figure 3A). However, degranulation in response to SP was significantly enhanced in PMCs generated from βArr2^−/−^ mice when compared with cells obtained from WT mice (Figure 3B). These findings suggest that β-arrestin2 expressed in MCs contributes to MrgprB2 desensitization in response to SP.

### 2.4. Mutation of a Highly Conserved Tyrosine Residue of MRGPRX2 (Y279A) Abolishes SP-Induced β-Arrestin Recruitment

**Figure 4:**
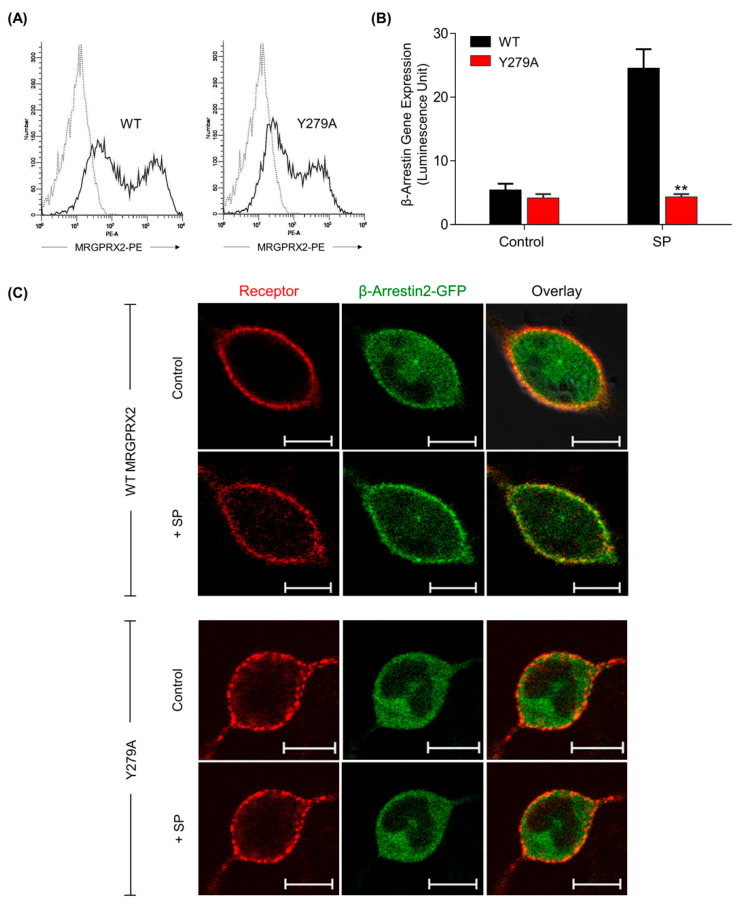
Y279A mutation of MRGPRX2 abolishes β-arrestin recruitment in response to SP. (**A**) HTLA cells were transiently transfected with cDNA encoding either WT-MRGPRX2 or its Y279A mutant, and cell surface receptor expression was determined by flow cytometry using PE-anti-MRGPRX2 antibody. (**B**) Cells were stimulated with SP (30 μM) for 16 h, and β-arrestin–mediated gene expression was measured. All data points are the mean ± SEM of at least three experiments. Two-tailed unpaired t-test was used. ** *p* < 0.01. (**C**) RBL cells co-expressing WT MRGPRX2 or Y279A and βArr2-GFP were stimulated with SP (30 μM) for 1 min and β-arrestin translocation was investigated by confocal microscopy. Scale bar = 10 μm.

The highly conserved NPxxY motif is important for GPCR activation and regulation [24,29]. Consistent with the findings in other GPCRs [29,40], the tyrosine residue located in the NPxxY motif of MRGPRX2 (Tyr^279^) has been previously shown to be essential for both SP-induced Ca^2+^ mobilization and degranulation [28]. We sought to determine whether this residue also mediates β-arrestin signaling. As we demonstrated that SP induced the β-arrestin pathway independently of G proteins, our initial hypothesis was that it might not contribute to the β-arrestin signaling. To assess this, we first constructed Y279A mutant in the MRGPRX2-Tango plasmid and generated transient transfectants in HTLA cells. We found that Y279A mutant showed a similar level of cell surface expression as the WT receptor (Figure 4A). Surprisingly, however, Y279A mutation resulted in a complete loss of β-arrestin-mediated gene expression in response to SP (Figure 4B).

One limitation of TANGO assay is that it measures β-arrestin-mediated gene expression after an overnight stimulation with agonist, thus not reflecting the typical β-arrestin recruitment that occurs rapidly within minutes [41]. Therefore, we utilized a green fluorescent protein-tagged β-arrestin2 plasmid (βArr2-GFP) to detect changes in SP-induced β-arrestin recruitment by confocal microscopy. βArr2-GFP were co-expressed transiently with either WT-MRGPRX2 or Y279A mutant in RBL-2H3 cells. As shown in Figure 4C, SP caused a rapid translocation (within a minute) of βArr2-GFP from cytoplasm to membrane in RBL-2H3 cells expressing the WT receptor. By contrast, this response was not observed in cells expressing Y279A mutant. These findings indicate that mutation of a highly conserved tyrosine residue of MRGPRX2 is required for SP-mediated β-arrestin recruitment.

### 2.5. Tyrosine Residue in MRGPRX2 (Y279) Is Required for SP-Mediated Receptor Internalization

**Figure 5:**
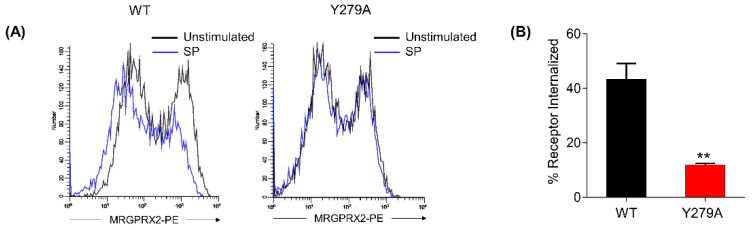
Y279A mutation of MRGPRX2 impairs SP-induced receptor internalization. (**A**) HTLA transiently expressing WT-MRGPRX2 or its Y279A mutant were stimulated with SP (30 μM) for 30 min, and the receptor internalization was determined by flow cytometry. Representative histogram for cell surface receptor expression before (black line) and after SP stimulation (blue line) are shown. (**B**) The percentage of receptor internalization after SP stimulation was calculated. All data points are the mean ± SEM of at least three experiments. Two-tailed unpaired *t*-test was used. ** *p* < 0.001.

We next examined the effect of Y279A mutation on SP-induced MRGPRX2 internalization. HTLA cells expressing the WT-MRGPRX2 exhibited reduced cell surface receptor expression following SP stimulation, as determined by flow cytometry, whereas SP was unable to trigger receptor internalization in cells expressing Y279A mutant (Figure 5A,B). 

**Figure 6:**
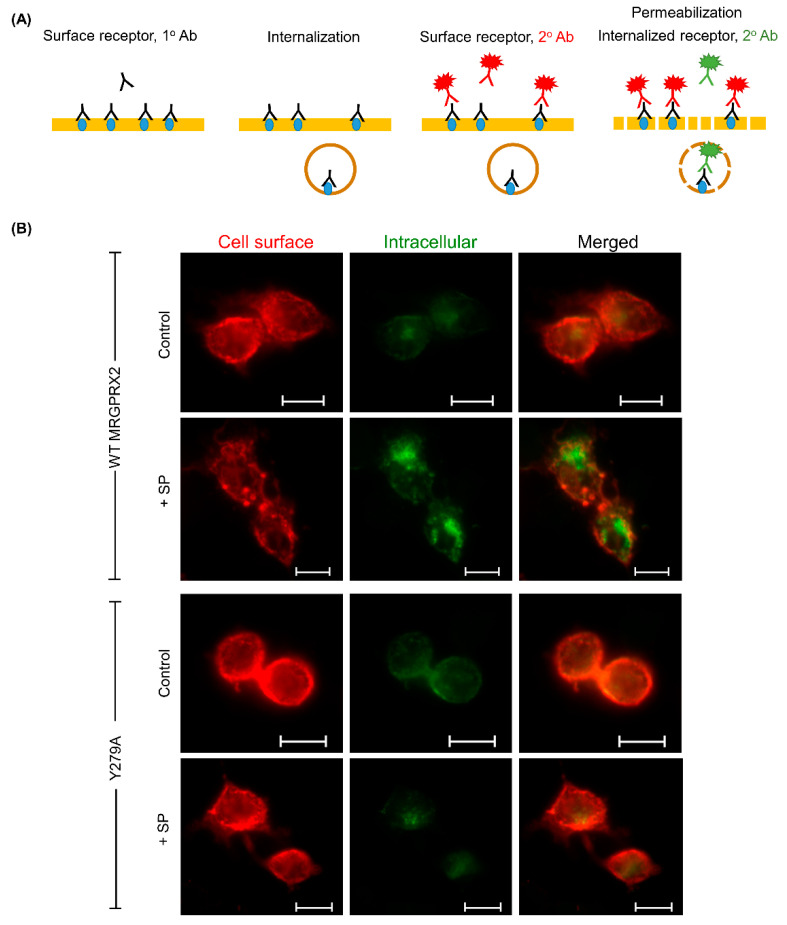
Y279A mutation of MRGPRX2 displays resistance to SP-induced receptor trafficking. (**A**) Schematic showing the dual-color labeling of cell surface and internalized receptors (modified from Carrodus et al., 2014 [42]). (**B**) Change in receptor trafficking was observed in RBL-2H3 cells expressing WT MRGPRX2 and Y279A mutant after SP stimulation (30 μM; 30 min). Scale bar = 10 μm.

We next performed an immunofluorescence study to confirm and visualize the MRGPRX2 receptor internalization in RBL-2H3 cells. The differential labeling of cell surface and internalized receptors technique was used [42]. For this, MRGPRX2 receptors expressed on the cell surface were labeled with 1^o^ antibody (unconjugated anti-MRGPRX2 antibody). Then, the receptor internalization was initiated by stimulating with SP for 30 min. The remaining surface receptors were labeled with Alexa Fluor 647-conjugated 2^o^ antibody (red). Cells were then permeabilized and the internalized receptors were labeled with Alexa Fluor 488-conjugated 2^o^ antibody (green) (Figure 6A). We found that, following SP stimulation, cell surface expression of WT-MRGPRX2 was significantly reduced and displayed a punctate pattern, while there was an increase in the number of internalized receptors, as indicated by an increased green signal. On the contrary, cells expressing Y279A mutant did not display receptor internalization and the expression of cell surface remained unchanged (Figure 6B). Taken together, these findings indicate that the conserved tyrosine residue in NPxxY motif of MRGPRX2 plays an important role in the receptor regulation and internalization upon stimulation by SP.

## 3. Discussion

Activation of MCs by SP has been implicated in the pathogenesis of neurogenic inflammation, pain, and itch via MRGPRX2 and its mouse counterpart MrgprB2 [3,4,43]. In addition to G proteins, most GPCRs have been shown to interact with β-arrestins for receptor desensitization and internalization. MRGPRX2 can undergo β-arrestin-mediated internalization in response to some, but not all agonists. For example, compound 48/80 and codeine act as balanced agonists for MRGPRX2 that activate both G proteins and β-arrestins, and thus can cause receptor internalization, which is associated with functional receptor desensitization [20,21]. On the contrary, host defense peptides (HDPs), such as cathelicidin LL-37 and angiogenic peptide AG-30/5C, activate only G proteins, but not β-arrestins (G protein-biased), and do not induce receptor desensitization and internalization [20,22,23]. Biased signaling has gained important therapeutic implications for several GPCRs [19]. However, whether SP is balanced or biased agonist for MRGPRX2 remains unknown. In this study, we demonstrated that SP serves as a balanced agonist for MRGPRX2 and that Tyr^7x53^ both contributes to G protein-dependent signaling for degranulation and promotes β-arrestin recruitment and MRGPRX2 internalization. Furthermore, β-arrestin2 negatively regulates MrgprB2-mediated MC degranulation in response to SP. Thus, targeting Tyr^7x53^ and β-arrestin2 could provide novel therapeutic modalities for modulating SP/MRGPRX2-mediated inflammatory diseases.

Based on the analysis of common activation pathway in 234 structures from 45 class A GPCRs, several conserved residues and key motifs have been identified that are involved in receptor activation and regulation [24]. Of these, the NPxxY sequence at the cytoplasmic end of the TM7 domain is one of the most highly conserved motifs among class A GPCRs. A study by Venkatakrishnan et al. suggested that Tyr^7x53^ in the NPxxY motif is essential for class A receptor activation and regulation [29]. We previously demonstrated that substitution of the corresponding tyrosine residue (Tyr^279^) in MRGPRX2 to Ala (Y279A) diminished SP-induced Ca^2+^ mobilization and degranulation responses [28]. Besides G protein signaling, mutations in the NPxxY motif of α_1B_-adrenergic and β_2_-adrenergic receptors have been associated with diminished agonist-mediated β-arrestin recruitment [44]. Here, we found that β-arrestin recruitment following SP stimulation was abolished in Y279A mutant when compared with the WT receptor, indicating that this tyrosine residue is also important for β-arrestin-mediated signaling via MRGPRX2.

The NPxxY motif has also been suggested as a common endocytic motif for GPCRs. The role of the tyrosine residue in the highly conserved NPxxY motif in receptor endocytosis and regulation has been established in certain GPCRs, including β_2_-adrenergic receptor [32], *N*-formyl peptide receptor [34], and NK-1R [35]. For example, mutation of the corresponding tyrosine residue of the β_2_-adrenergic receptor to alanine (Y326A) abolishes agonist-induced receptor phosphorylation, internalization, and desensitization [32,45]. Similarly, formyl peptide receptor mutation Y301A results in a complete loss of agonist-induced receptor internalization [34]. However, while this conserved tyrosine residue appears to be essential for agonist-induced receptor internalization of some GPCRs, it is not required for the internalization of angiotensin II receptor [46,47] or gastrin-releasing peptide receptor [48]. Thus, it is possible that the role of a highly conserved tyrosine residue in the NPxxY motif on agonist-induced receptor internalization is receptor specific. Here, we found that MRGPRX2 Y279A mutation impaired receptor internalization in response to SP and caused retention of receptors at the cell surface. These findings suggest that the tyrosine residue in the NPxxY motif of MRGPRX2 is important for SP-induced receptor internalization. It is uncertain whether Tyr^279^ affects MRGPRX2 phosphorylation, and thus contributes to receptor internalization. It will be important to further investigate the effect of this tyrosine residue and NPxxY motif on the receptor phosphorylation. Furthermore, the mechanistic pathway responsible for MRGPRX2 internalization should be further identified.

It is worth noting that, in this study, we mostly utilized HTLA and RBL-2H3 cell lines transfected with human MRGPRX2 to investigate the effects of SP on MRGPRX2 regulation. While recent evidence demonstrates that ectopically expressed MRGPRX2 retains its authentic mechanisms for MC activation and degranulation as of human MCs endogenously expressed MRGPRX2 [37], further studies in human-derived MCs are warranted to confirm these findings.

In summary, this study extends our previous observations on the pivotal role of tyrosine residue Tyr^279^ in the NPxxY motif of MRGPRX2 on G protein activation [28]. The data presented herein demonstrate the novel finding that this single Tyr^279^ residue contributes to both G protein and β-arrestin signaling in response to SP. Substitution of Tyr^279^ to alanine (Y279A) abolishes SP-induced MC degranulation, β-arrestin recruitment, and MRGPRX2 internalization. This tyrosine residue may contribute to the regulation of MRGPRX2 activation and internalization, presumably by maintaining receptor conformation, thus controlling G protein coupling and activation as well as the internalization process. These findings have an important clinical implication as individuals harboring this mutation may become resistant to developing neurogenic inflammation and inflammatory diseases.

## 4. Materials and Methods

### 4.1. Materials

All cell culture and Lipofectamine 2000 transfection reagents were obtained from Invitrogen (Gaithersburg, MD, USA). Amaxa Nucleofector kit (Kit V) was purchased from Lonza (Gaithersburg, MD, USA). Phycoerythrin (PE)-conjugated (Cat.#359004) and purified unconjugated anti-MRGPRX2 antibodies (Cat.#359002) were from BioLegend (San Diego, CA, USA). Donkey anti-mouse Alexa Fluor 488 (Cat.#A21202) and 647 (Cat.#A31571) conjugated IgG secondary antibodies were from Invitrogen (Gaithersburg, MD, USA). p-nitrophenyl-N-acetyl-β-D-glucosamine (PNAG) was from Sigma-Aldrich (St. Louis, MO, USA). Fura-2 acetoxymethyl ester was from Abcam (Cambridge, MA, USA). SP was purchased from AnaSpec (Fremont, CA, USA). Pertussis toxin (PTx) was from List Biological Laboratories (Campbell, CA, USA). Plasmid encoding hemagglutinin (HA)-tagged human MRGPRX2 in pReceiver-MO6 vector was obtained from GeneCopoeia (Rockville, MD, USA). MRGPRX2 Y279A mutant in HA-tagged plasmid was reported previously [28]. MRGPRX2-Tango plasmid (Addgene no. 66440) was a gift from Dr. Bryan Roth. MRGPRX2 Y279A mutant in Tango plasmid was generated by Penn Genomics Analysis Core (Philadelphia, PA, USA).

### 4.2. Mice

C57BL/6 (wild-type; WT) and β-arrestin2 knockout (βArr2^−/−^) mice were obtained from the Jackson Laboratory (Bar Harbor, ME, USA). Mice were housed in pathogen-free cages on autoclaved hardwood bedding. Eight-to-twelve-week-old male and female mice were used in this study. All experiments were approved by the Institutional Animal Care and Use Committee of the University of Pennsylvania.

### 4.3. Cell Culture

Rat basophilic leukemia (RBL-2H3) cells were maintained as monolayer cultures in Dulbecco’s modified Eagle’s medium (DMEM) supplemented with 10% FBS, L-glutamine (2 mM), penicillin (100 IU/mL), and streptomycin (100 µg/mL) at 37 °C with 5% CO_2_ [49]. RBL-2H3 cells stably expressing MRGPRX2 (RBL-MRGPRX2) were used and maintained similarly in the presence of G-418 (1 mg/mL) [22,50].

HTLA (engineered HEK-293T cells stably expressing a β-arrestin2–tobacco etch virus fusion gene) cells and HTLA cells stably expressing MRGPRX2-Tango (HTLA-MRGPRX2) were maintained in DMEM supplemented with 10% FBS, l-glutamine (2 mM), penicillin (100 IU/mL), streptomycin (100 µg/mL), hygromycin B (200 µg/mL), puromycin (5 mg/mL), and G-418 (500 µg/mL) [25,51].

Peritoneal MCs (PMCs) were purified from WT and βArr2^−/−^ mice as described previously [39]. Briefly, the peritoneal cavity was lavaged with 10 mL of HBSS supplemented with 3% FCS and 10 mM HEPES. The cells were cultured in Iscove’s modified Dulbecco’s medium (IMDM) supplemented with 10% FCS, murine IL-3 (10 ng/mL), and murine SCF (30 ng/mL). After 48 h, non-adherent cells were removed and adherent cells were cultured in fresh medium for an additional 10–14 days. Suspension cells were then determined for MC receptor expression and function and were used for experiments as PMCs.

### 4.4. Generation of Cells Transiently Expressing WT MRGPRX2 and Its Variant

RBL-2H3 cells (2 × 10^6^) were transiently transfected with 2 µg of HA-tagged plasmid using the Amaxa Nucleofector Device and Amaxa Kit V according to the manufacturer’s protocol. Cells were used within 16–20 h after transfection [27].

For HTLA cells transiently expressing WT MRGPRX2 or its missense variant, cells (1 × 10^6^ cells per well) were plated in a six-well plate in antibiotic-free medium (DMEM supplemented with 10% FBS and L-glutamine) and incubated overnight at 37 °C with 5% CO_2_. The following day, cells were transfected with 2 µg of MRGPRX2 or its missense variants in Tango plasmids using the Lipofectamine 2000 DNA transfection reagent according to the manufacturer’s protocol. Cells were incubated overnight at 37 °C with 5% CO_2_ in antibiotic-free medium and were used within 16–48 h after transfection [25].

### 4.5. Receptor Expression and Internalization Using Flow Cytometry

Cells expressing either WT MRGPRX2 or its mutants (5 × 10^5^) were stimulated with either SP or buffer for the indicated time at 37 °C. Cells were washed twice with ice-cold FACS buffer (PBS containing 2 % FCS and 0.02% sodium azide) and incubated with PE-conjugated anti-MRGPRX2 antibody for 30 min at 4 °C in the dark. Cells were then washed with FACS buffer and fixed in 1.5% paraformaldehyde. Cells were acquired using a BD LSR II flow cytometer (San Jose, CA, USA) and MRGPRX2 expression was analyzed using WinList software, version 8.

### 4.6. Degranulation 

The degranulation was measured by β-hexosaminidase release as described previously [49]. Briefly, RBL-2H3 cells (5 × 10^4^ cells) or PMCs (1 × 10^4^ cells) were seeded into a 96-well, white, clear-bottom cell culture plate and incubated overnight in a 37 °C incubator with 5% CO_2_. Cells were then washed twice and suspended in a total volume of 50 μL HEPES buffer containing 0.1% bovine serum albumin (BSA). Experimental groups were stimulated with SP for 30 min at 37 °C. Cells without treatment were designated as controls. To determine the total β-hexosaminidase release, unstimulated cells were lysed in 50 μL of 0.1% Triton X-100. Aliquots (20 μL) of supernatants or cell lysates were incubated with 20 μL of 1 mM p-nitrophenyl-N-acetyl-β-D-glucosamine (PNAG) for 1 h at 37 °C. The reaction was stopped by adding 250 μL of stop buffer (0.1 M Na2CO3/0.1 M NaHCO3). The β-hexosaminidase release was assessed by measuring absorbance at 405 nm using Versamax microplate spectrophotometer (San Jose, CA, USA).

### 4.7. Transcriptional Activation Following Arrestin Translocation (TANGO) Assay 

HTLA cells expressing either WT MRGPRX2 or its variants (5 × 10^4^ cells per well) were plated into a 96-well plate in triplicates in a total volume of 160 μL antibiotic-free medium and incubated for 6 h at 37 °C to allow attachment. After 6 h, the medium was aspirated, and cells were incubated with MRGPRX2 ligands in 160 μL antibiotic-free medium for additional 16 h at 37 °C. The medium and ligands were then aspirated and 100 μL of Bright-Glo solution (Promega) was added to each well. Relative luminescence unit was measured in a Thermo Labsystems Luminoskan Ascent 392 Microplate Luminometer [20,25].

### 4.8. β-Arrestin Translocation by Live Imaging Confocal Microscopy

RBL-2H3 cells were co-transfected with green fluorescent protein-tagged β-arrestin2 plasmid (βArr2-GFP) and WT-MRGPRX2 or its mutants with a ratio of 3:1 for β-arrestin2/receptor using Amaxa Kit V as described above. Transfected cells were plated onto a 35 mm glass bottom dish. Cell surface receptor was determined by incubating with purified anti-MRGPRX2 antibody, followed by Alexa Fluor 647-conjugated secondary antibody. Cells were then stimulated with SP and live images of β-arrestin2 translocation as indicated by green fluorescence images were collected using Nikon A1R confocal microscope.

### 4.9. Receptor Trafficking Using Immunofluorescence Microscopy

Receptor trafficking after SP stimulation was modified from previously described antibody feeding assay [42]. RBL-2H3 cells expressing either WT-MRGPRX2 or its variants were plated onto sterilized glass coverslips (2 × 10^5^ cells/12 mm diameter coverslip in a 24-well plate) and incubated overnight at 37 °C with 5% CO_2_ to allow attachment. Cells were rinsed with PBS and blocked with blocking buffer (PBS with 2% BSA) for 30 min at room temperature. Primary antibody incubation was performed using purified anti-MRGPRX2 antibody (1:250 dilution) for 1 h at 4 °C to label the cell surface expressed receptors. Cells were then stimulated with SP for 30 min at 37 °C to allow internalization, followed by being fixed with 4% paraformaldehyde for 15 min at 4 °C. Labeled surface receptors were detected by incubating with saturated Alexa Fluor 647-conjugated secondary antibody (red) for 1 h at 4 °C. Cells were then permeabilized using 0.2% Triton X-100 in blocking buffer for 30 min and internalized receptors were detected by incubating with Alexa Fluor 488-conjugated secondary antibody (green) for 30 min at 4°C. Then, cells were mounted onto the glass slides using ProLong Gold Antifade mounting medium (Invitrogen) and images were visualized using a Nikon Eclipse N*i* microscope.

### 4.10. Statistical Analysis 

Data shown are mean ± standard error of the mean (SEM) values derived from at least three independent experiments. GraphPad Prism scientific software version 6.07 was used for statistical analysis. Statistical significance was determined using unpaired two-tailed t-test and one- or two-way ANOVA. Differences were considered as statistically significant at a value * *p* < 0.05, ** *p* < 0.01, *** *p* < 0.001, and **** *p* < 0.0001.

## Data Availability

No new data were created or analyzed in this study. Data sharing is not applicable to this article.
